# Influence of Impregnation with Modified Starch of a Paper Core on Bending of Wood-Based Honeycomb Panels in Changing Climatic Conditions

**DOI:** 10.3390/ma15010395

**Published:** 2022-01-05

**Authors:** Michał Słonina, Dorota Dziurka, Marta Molińska-Glura, Jerzy Smardzewski

**Affiliations:** 1 Department of Furniture Design, Faculty of Forestry and Wood Technology, Poznan University of Life Sciences, Wojska Polskiego 28, 60-637 Poznan, Poland; 2 Department of Mechanical Wood Technology, Faculty of Forestry and Wood Technology, Poznan University of Life Sciences, Wojska Polskiego 28, 60-637 Poznan, Poland; dorota.dziurka@up.poznan.pl; 3 Department of Economics and Forest Technology, Faculty of Forestry and Wood Technology, Poznan University of Life Sciences, Wojska Polskiego 28, 60-637 Poznan, Poland; marta.glura@up.poznan.pl

**Keywords:** honeycomb panels, starch, impregnation, climatic conditions, strength, stiffness, energy absorption

## Abstract

The main objective of the study was to determine the effect of impregnation of the paper core with acetylated starch on the mechanical properties and absorbed energy in the three-point bending test of wood-based honeycomb panels under varying temperatures and relative air humidity conditions. Nearly six hundred beams in various combinations, three types of facings, three core cells geometries, and two paper thicknesses were tested. The experiment results and their statistical analysis prove a significant relationship between the impregnation of paper with modified starch and mechanical properties. The most effective in absorbing energy, the honeycomb panels, consisted of a core with a wall thickness of 0.25 mm and a particleboard facing.

## 1. Introduction

Production of paper products in 2020 reached the level of 420 million tons. Compared to 1980, this means an increase of 250% [1]. Invariably, for over 2000 years, the paper has been produced mainly from cellulose fibers [2]. It is assumed that the life cycle of cellulose fiber in Europe has an average of 3.5 times its use [3,4], although it is possible up to 6 times. Each cycle of paper reuse reduces its quality [5]. Out of all known paper types, “Kraft liner” is characterized by the best value for money. The quality of the paper means its high mechanical strength to tearing, bending, compression, and resistance to moisture [6]. Kraft liner is made by chem ical defibering with at least 80% virgin fibers. It is widely used as a packaging material [7]. In the range of moisture content of the paper from 0% to the fiber saturation point (about 23%), its mechanical properties decrease even by 50% [8,9,10]. The pulp and paper industry still uses various preservation methods against hygroscopy and shrinking of paper, including impregnation methods. Pohl [11] described the influence of the paper’s sizing on the reduction of tensile strength.

This does not mean that waterproof paper cannot be made. However, depending on the chosen path, the process can be more or less complicated and time consuming. The simplest solution is lamination with petroleum-based or aluminum foils. The composite obtained in this way is completely waterproof and has higher mechanical strength, especially for tearing and penetration [12]. Lamination is also an effective barrier against gas penetration. However, it should be remembered that the edges of this paper composite remain hydrophilic. Another solution is to use chemicals while still producing the paper web. Mainly to obtain covalent bonds between cellulose fibers. Urea-formaldehyde (UF), melamine-formaldehyde (MF) and polyamide-epichlorohydrin (PAE) resins are used as an additive to the pulp or preformed paper.

The most commonly used neutral sizing agents are softwood extracts and alkyl ketene dimers (AKD), and alkenyl succinic acid anhydride (ASA) [13,14]. In recent years, efforts have been made to develop environmentally friendly substances that increase the hydrophobic properties of cellulose fibers. We are talking about plant proteins or starch [15,16]. Both cellulose and starch are homoglycans and are the most abundant polysaccharides in nature [17]. Starch is the second most used improver in the pulp and paper industry, right after clay fillers. The usual addition to pulp is in the range of 2–4% [17,18]. Its presence increases the mechanical resistance of the paper to tearing, improves the quality of prints, and most of all increases the resistance to moisture by filling the pores in the cellulose fiber mesh [19]. In 2009, modified starch accounted for 66% of the total volume of starch used against the sizing effect [18]. There are enzymatic, thermal, and chemical modifications [5,20]. In a chemical acetylation process, a hydrogen atom in the hydroxyl (OH) group is replaced by an acetyl group. Starch has three OH groups, so its maximum degree of substitution (DS) is 3. The higher the degree of substitution, the greater the hydrophobicity [21]. However, it should be remembered that the strength of paper precisely increases thanks to the hydrogen bonds between cellulose and starch [15]. As demonstrated by Larotonda et al. [22], acetylation at the DS 1.2–1.7 level provides the best balance between paper strength and resistance to moisture while maintaining the possibility of biodegradability. The production of hydrophobic Kraft liner paper is, therefore, a difficult and complex task. Serious problems are also encountered concerning obtaining water resistance of recycled paper of the “testliner” type. It is recycled, so the cellulose pulp can contain almost all the additives mentioned so far and many more from impurities.

As reported by European and global organizations monitoring the pulp and paper industry, in 2018, more than half of the global paper production was constituted by testliner [1,23]. This paper is mainly used to produce the recycled corrugated panel, both for a sinusoidal core, where its transversal shear properties are significant [24,25,26,27,28], and for facings, where its properties are related to edge crush resistance are important [29,30,31]. In addition, this paper is used to produce paper fillings (honeycomb cores), used in the production of a three-layer lightweight panel. For the same reasons why recycled paper processing is growing dynamically, the share of light furniture panels in the furniture industry is also growing.

Light wood-based honeycomb panels are widely used in the production of doors [32]. In the 1990s, the technology was adapted to the needs of the furniture industry [33,34,35]. However, a significant limitation of the widespread use of lightweight panels in the furniture industry is their low stiffness and strength, compared to classic wood materials, such as particleboard, MDF board, or plywood [36,37,38,39,40]. However, these panels are distinguished by an attractive quality factor [41,42]. For this reason, more and more manufacturers of furniture ready for self-assembly (RTA), made of honeycomb panels, dynamically develop the e-commerce market [43,44,45,46]. For these products to be safe in terms of construction, research was carried out on the rheology and strength of the constituent materials of light honeycomb sandwich panels under changing climatic conditions [47,48,49,50] and the properties of wood-based furniture panels [51,52,53,54,55]. Moreover, research works on methods of securing wood-based honeycomb panels against the destructive effects of variable temperature and air humidity [53,56,57,58,59].

Composites based on thin-walled cores are also a sought-after products by the packaging industry to protect valuable loads [60]. Their task is to absorb impact energy [61,62], an indispensable element of the global flow of goods, using diverse and complementary means of transport by land, sea, and air. International transport of goods also means highly different climatic conditions, so it is crucial to properly design thin-walled structures to maintain their ability to absorb energy [63,64] throughout its life cycle. The more energy the composite can absorb, the more effectively it can protect a product against the outside load.

However, the authors’ best knowledge shows that the influence of the hydrophobic impregnation of the paper core with modified starch on the mechanical properties of the honeycomb panel has not been investigated so far. Acquiring new knowledge enables learning about the effectiveness of securing furniture elements against the effects of variable high temperature and air humidity. This knowledge will allow the rational design of furniture intended for use in tropical or subtropical climates. It is also justified in the changing demographic structure of the world. By 2050, half of the world’s population will live in a tropical climate [65]. Until then, the number of users of honeycomb panel furniture resistant to tropical climatic conditions will increase.

The study aimed to determine the influence of the impregnation of paper with modified starch, the shape and size of the hexagonal core cells obtained from the impregnated paper, and the facing material on the bending of the wood-based lightweight honeycomb panels under changing temperature and relative air humidity conditions.

## 2. Materials and Methods

### 2.1. The Shape of Honeycomb Cells

**Figure 1:**
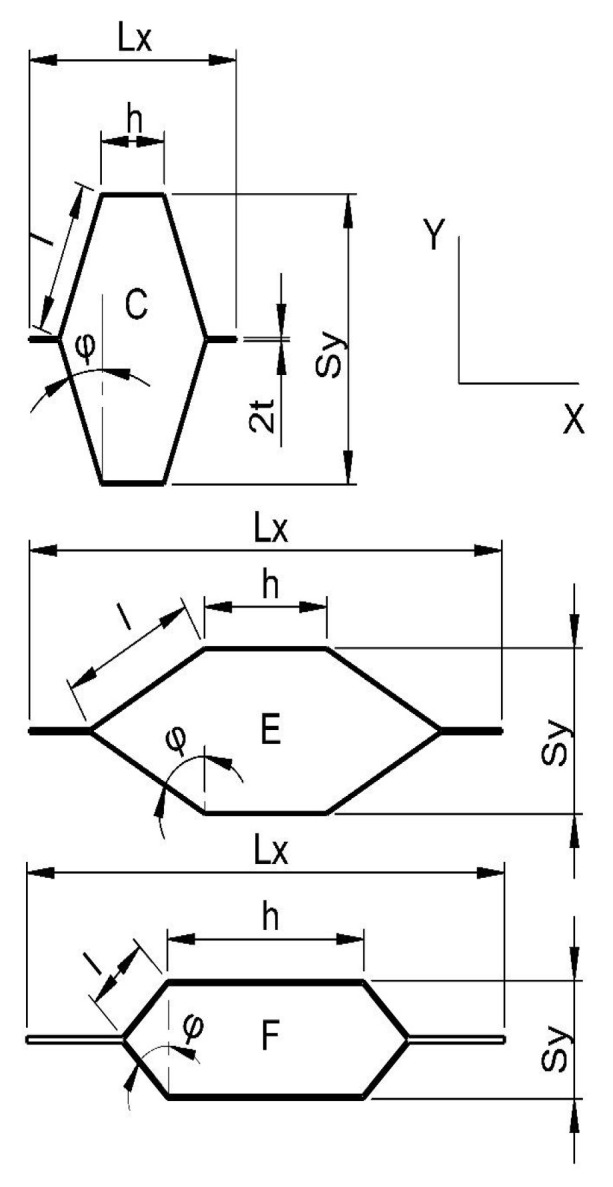
The shape of cells used for research.

A series of scientific publications [66,67,68] describes in detail the method of selecting cell geometry and its production processes. On this basis, keeping the previously used determinations, cores with cells geometry C, E, F were selected for the tests (Figure 1). The cores of the cells of type C and E are made of paper testliner having a thickness of 0.15 mm and a weight of 123 g/m^2,^ and the cores with F cells were made of 0.25 mm thick paper with a grammage of 134 g/m^2^. Such a selection of papers was not left to chance. The F-cell paper thickness was determined by static numerical optimization with the Monte-Carlo method [66]. They were assuming that the linear modulus is maximized and the relative cell density is minimized. C and E cores are made of the most used paper in the furniture industry [66,69]. The decision to use cores based on C, E, F cells to create light furniture panels results from a thorough analysis of the elastic constants of individual cores carried out in the publication of Słonina et al. [68]. The exact dimensions of individual types of cells and their relative density are presented in Table 1.

The testliner paper was produced by the HM Technology company (HM Technology, Brzozowo, Poland). For cell formation, non-impregnated (N) and impregnated (S) papers were prepared with a 10% aqueous solution of modified acetylated starch (S) (patent number P.430486). Depending on their thickness and impregnation, these papers were marked with the symbols 15N, 25N, 15S, 25S, respectively. The paper [68] presents in detail the method of paper impregnation, forming cells, and obtaining cores. In addition, the results illustrating the elastic properties of paper, which were determined following the PN-EN ISO 1924-2 standard [70], are also presented. For the sake of clarity of this work, Table 2 is summarized by providing the module of linear elasticity MOE (MPa), the module of rupture MOR (MPa) Poisson’s ratio, and the maximum breaking force X and Y direction of the material orthotropy, respectively. Table 2 also shows the elastic properties of the materials used to produce the facings of the honeycomb panels. These properties were determined following ISO 13061-6: 2014 [71].

### 2.2. Honeycomb Manufacturing and Testing

**Figure 2:**
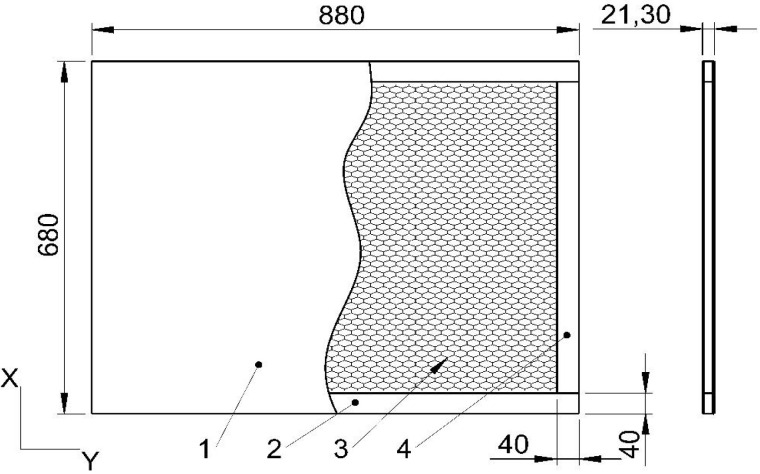
Dimensions and structure of the honeycomb panel sheet: 1—facing, 2—honeycomb core, 3—horizontal stile, 4—vertical stile. Panels thickness equal to 20.3 mm, 21.3 mm, 22.3 mm for H20, H25, P30, respectively.

The testliner paper was produced by the HM Technology company (HM Technology, Brzozowo, Polska). Non-impregnated papers were prepared for cell formation. The facing material was selected from wood-based materials that retain the ability to be reused in recycling processes. Thus, a 3.0 mm thick particleboard (P30) covered on one side with melamine paper (Egger, Rion-des-Landes, France) [72], a 2.5 mm thick high-density fiberboard (H25) (IKEA Industry, Orla, Polska) [73], and high-density fiberboard with a thickness of 2.0 mm (H20) (HOMANIT, Karlino, Polska) [74] was used for the tests. On nondecorative surfaces of the same type of facing panel, an adhesive PVAc Woodmax FF12.47 class D2 from Synthos Adhesives (Oswiecim, Poland) was applied in an amount of about 110 g/m^2^. In the next step, along the circuit of bottom facing, a particleboard frame with a thickness of 16.1 mm was created, and a paper core with a thickness of 16.3 mm was placed inside it. Finally, the whole sandwich was closed by the second facing sheet. The assembly process was carried out in an Orma Macchine NPC/DIGIT 6/90 25 × 13 hydraulic press (Bergamo, Italy) for 25 min under a pressure of 0.7 MPa. For each type of impregnated and non-impregnated paper, the cell type and the type of facing six 16 mm thick panels were made with dimensions as shown in Figure 2. A total of 54 panels were manufactured.

The panels were seasoned in laboratory conditions until a constant mass of samples was obtained, which proved that they maintained the hygroscopic equilibrium. After this time, the panels were cut into beams 50 mm wide and 20 times their length, plus an allowance of 50 mm. The beams were obtained from the central part of the formed slab so that the samples did not contain stiles. The produced beams were divided into two groups of equal numbers. The first group of beams was stored in dry conditions (D), i.e., in the climate of the production hall at the temperature T = 25 °C and relative air humidity H = 45%, while the second group of beams was stored in a climate similar to tropical (W), i.e., at the temperature T = 28 °C and relative air humidity of H = 85%, until the mass of the samples stabilizes. The selected air temperature and relative humidity complied with EN 318 (2002) requirements and were used as variable factors in the works [68,75,76].

**Figure 3:**
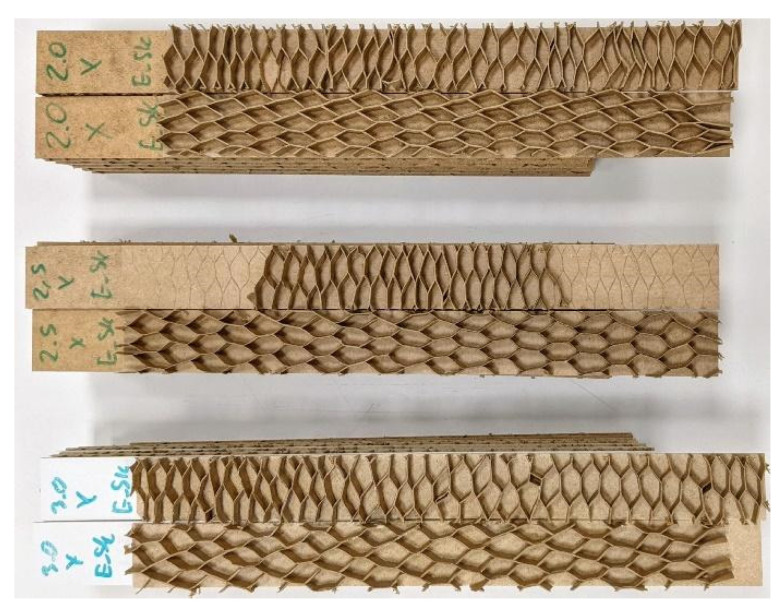
Examples of beams selected for testing.

For the selected types of cells (3), the impregnated and non-impregnated paper (2) used, the facings (3), the direction of X, Y orthotropy (2), climatic conditions (2), assuming eight repetitions, in total 576 pieces of beams were prepared for testing (Figure 3). Table 3 presents exemplary determinations for individual types of samples produced. According to the method of marking the beams made of C-type cells, the markings for the remaining E, F-type cells were used, as exemplified in the last row of Table 3.

**Figure 4:**
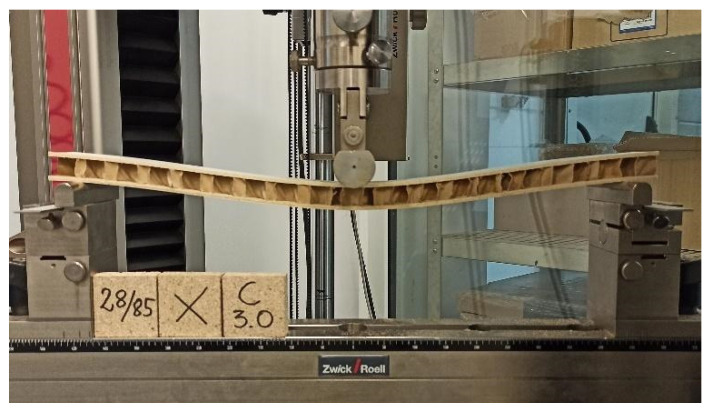
Test stand.

Then the beams were subjected to three-point bending (Figure 4) according to the EN 310 [77] standard on the Zwick Z100 testing machine (Zwick GmbH, Ulm, Germany). During the tests, the value of the force was recorded with an accuracy of 2 N and the deflection of the beams in the direction of the force with an accuracy of 0.01 mm. In addition, damage to the beams was recorded using a Samsung SM20E digital camera (SM20E, Samsung, Korea).

### 2.3. Mechanical Properties and Energy Absorption

**Figure 5:**
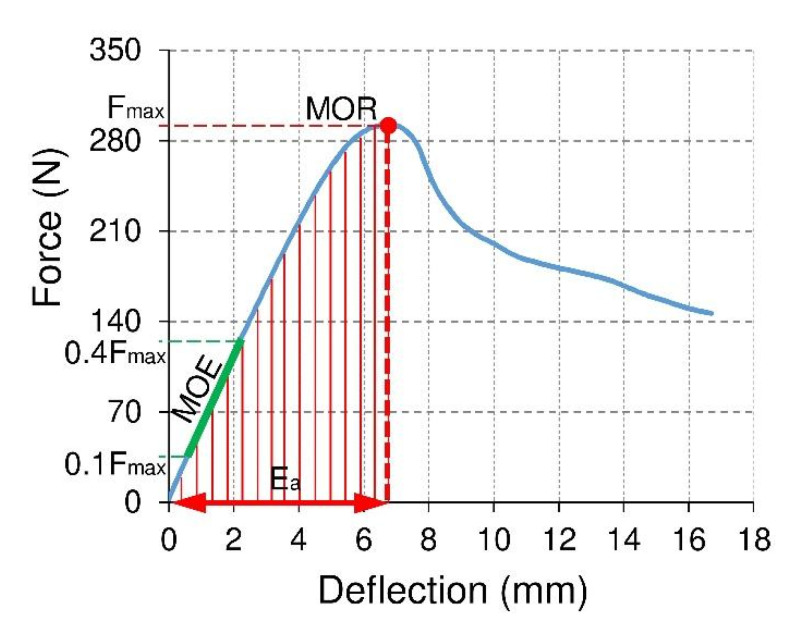
An example of a force-deflection relationship for examined beams made of honeycomb panels *(*MOR—modulus of rupturę, MOE—modulus of elasticity, $F_{\max}$—fracture force, $E_{a}$—absorbed energy).

Figure 5 shows an example of a curve expressing the relation of force and deflection for the tested samples. Based on the measured values of the maximum forces causing the sample failure $F_{\max}$ (N) modulus of rupture MOR (MPa) was calculated following the EN 310 standard from the dependence:

(1) $$\mathrm{MOR}=\frac{3F_{\max}L^{3}}{2{\mathrm{bd}}^{3}}$$

where $F_{\max}$ is the force at the fracture point (N), L=20 d is the length of the support span (mm), d is the thickness of the beam (mm), b is the width of the beam (mm).

On the other hand, based on the relationship of force and deflection in the rectilinear range, the linear elasticity modulus MOE (MPa) was calculated by the EN 310 standard, from the following equation:

(2) $$\mathrm{MOE}=\frac{\left(0.4F_{\max}-0.1F_{\max}\right)L^{3}}{48\left(f_{0.4\mathrm{Fmax}}-f_{0.1\mathrm{Fmax}}\right)I_{s}}$$

where $f_{0.4\mathrm{Fmax}\;},{\;f}_{0.1\mathrm{Fmax}}$ is the beam deflection in mm for a load equal to $0.4F_{\max},0.1F_{\max}$ (N), $I_{s}=\frac{{\mathrm{bd}}^{3}}{12}$ is the moment of inertia (mm^4^).

The individual beams were made of thin-walled elements. Therefore, it was concluded that they should be excellent energy-absorbing structures because they can fail with relatively little force. To obtain comparable calculation results, it was decided to count the absorbed energy only to obtain the maximum breaking force of the beam.

By integrating the function expressing the dependence of force on deflection (Figure 5), it is possible to calculate the absorbed energy from the equation [60,61,62,63,64]:

(3) $$E_{a}=\int_{f1}^{f2}\mathrm{Fdf}$$

where $f_{1\;},{\;f}_{2\;},$ is the lower and upper integration limits for the deflection, respectively f. However, due to the inability to determine the exact functions describing the force F deflection relationship f for each tested sample, it was decided to perform graphical integration. For this purpose, the integration interval was divided $<f_{1\;},{\;f}_{2}>$ into segments $\Delta f_{\;}=0.1$ mm, on which it was assumed that the force F has a constant value. Therefore, the absorbed energy for deflection equal to 10 mm was calculated from the equation:

(4) $$E_{a}=\overset{f2=10}{\underset{f1=0}{\sum }}F\Delta f$$

## 3. Results and Discussion

The influence of starch impregnation on selected properties of honeycomb panels turned out not to be obvious. Therefore, this part of the work decided to present only the observed quantitative differences. In the other part of the study, a detailed statistical analysis was prepared to show the qualitative relationships and the impact of all selected variable factors on the properties of the tested honeycomb panels.

### 3.1. Effect of Impregnation on the Panel’s Strength

**Figure 6:**
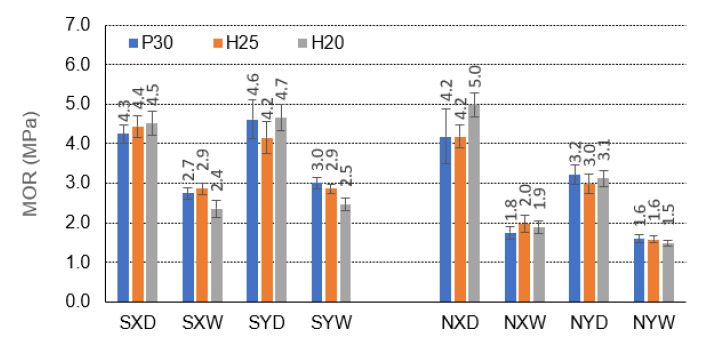
Illustration of the MOR relationship of honeycomb panels with a core of C-cells. Whiskers represent standard deviations.

**Figure 7:**
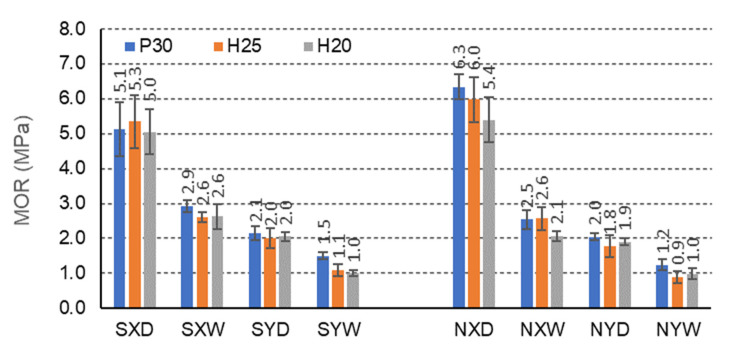
Illustration of the MOR relationship of honeycomb panels with an E-cell core. Whiskers represent standard deviations.

**Figure 8:**
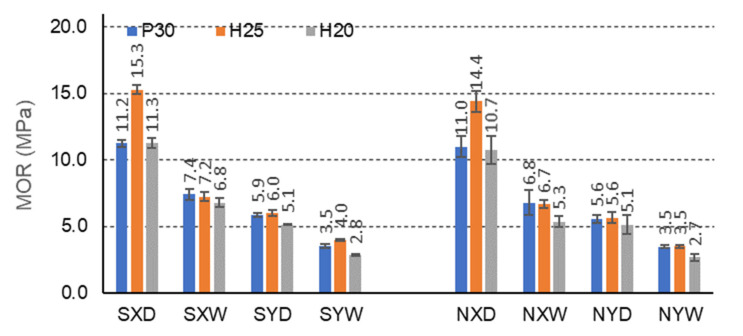
Illustration of the MOR relationship of honeycomb panels with F-type core. Whiskers represent standard deviations.

Figure 6, Figure 7 and Figure 8 illustrate the effects of starch impregnation, type of material, the direction of orthotropy, and climatic conditions on the MOR of honeycomb panel with a core of different cells (C,E,F). The summary shows that the highest MOR (15.3 MPa) was observed among the beams with starch impregnated F-cells for the X orthotropy direction in dry conditions (FSH25XD). Conversely, the lowest MOR (0.9 MPa) occurred in the case of beams with non-impregnated E-type cells for the Y orthotropy direction in tropical conditions (ENH25YW).

In the group of beams with C-cells, under all climatic conditions D, W, the starch impregnation improves the MOR of the beams for each case of the facing used and the orthotropy direction (Figure 6). Only CNP30XD beams with P30 facings in dry conditions and the X orthotropy direction show higher strength (5.0 MPa) than the corresponding CSP30XD beams impregnated with starch (4.5 MPa). It should be noted here that the increase in MOR for beams with impregnated cores compared to beams with non-impregnated cores is from 2.3 to 46.7%. For beams with P30, H25, and H20 facings, in dry conditions D and for the direction of orthotropy X, the bending strength increases by 2.3%, 4.5%, and −11.1%, respectively, and in tropical conditions W by 33.3%, 31% and 20.8, respectively. Note that a negative value indicates the opposite tendency to increase—i.e., decrease. For beams with the same facings P30, H25 and H20, in dry conditions D, and for the direction of orthotropy Y, the bending strength increases by 30.4%, 28.6%, and 34.0%, respectively, and in tropical conditions W by 46.7%, 44.8%, and 40.0%, respectively. It is also significant that for C-type cells, there was no clear difference in the strength of the beams in the X and Y orthotropy directions greater by −7.0%, 4.5%, and −4.4% in relation to the strength in the Y direction, and in tropical conditions W by 11.1%, 0.0%, and −4.2%, respectively. For non-impregnated beams, these relationships are significantly different. For beams with P30, H25, and H20 facings, in dry conditions D, the bending strength in the direction of X orthotropy is respectively higher by 23.8%, 28.6%, and 38.0% concerning the strength in the Y direction and tropical conditions W, respectively 11.1%, 20.0%, and 21.1%. Thus, the effect of impregnation on the strength of beams with C-cells is visible, but also on the change of this strength depending on the direction of orthotropy. After impregnation, the influence of the direction of orthotropy on the strength of the beams decreased significantly.

For beams with E-cells, the effect of impregnation is not so pronounced (Figure 7). Beams with P30 facing, not impregnated, in dry conditions D, and the orthotropy direction X (6.3 MPa) show the highest bending strength. In dry conditions D, impregnation with starch significantly reduces the MOR of beams with P30, H25, H20 facings, for the X orthotropy direction, compared to analogous non-impregnated beams by 23.5%, 13.2%, 8.0%, respectively. In tropical conditions, W increases this strength by 13.8%, 0.0%, and 19.2%, respectively. For beams with the same facings P30, H25 and H20, in dry conditions D, and for the direction of orthotropy Y, the bending strength increases by 4.8%, 10.0%, and 5.0%, respectively, and in tropical conditions W, by 20.0%, 18.2%, and 0.0%, respectively. It is also noticeable that clear differences in the strength of the beams in the X and Y orthotropy directions were observed for E-type cells, by 58.8%, 62.3% and 60.0% of the strength in the Y direction, and in tropical conditions by 48.3%, 57.7% and 61.5%, respectively. For non-impregnated beams, these relationships are very similar. For beams with P30, H25, and H20 facings, in dry conditions D, the bending strength in the direction of X orthotropy is, respectively, 68.3%, 70.0%, and 64.8% greater concerning the strength in the Y direction, and in tropical conditions W, respectively 52.0%, 65.4%, and 52.4%. Moreover, in this case, the influence of impregnation on the strength of beams with E-cells is visible. The presented results also illustrate the effect of large and slightly changing orthotropy of the tested beams. It can be assumed that the effect of impregnation on the change of orthotropic properties of the panel is significant.

The F-cell beams also show marked strength differences due to the impregnation of the paper (Figure 8). Beams with H25 facing, impregnated, in dry conditions D, and for the orthotropy direction X (15.3 MPa) show the highest bending strength. Beams with H25 facing, not impregnated, in dry conditions D achieve slightly lower strength for the same direction of orthotropy (14.4 MPa). In dry conditions D, impregnation with starch slightly improves MOR of beams with P30, H25, H20 facings, for the X orthotropy direction, compared to non-impregnated beams by 1.8%, 5.9%, 5.3%, respectively, and in tropical conditions by 8.1%, respectively, 6.9% and 22.1%. Beams with the same facings P30, H25, and H20, in dry conditions D, and for the orthotropy direction Y show greater bending strength by 5.1%, 6.7%, and 0.0%, respectively, and in tropical conditions W by 0.0%, 12.5%, and 3.6%, respectively. As in the case of beams with E-cells, clear differences in the strength of the beams in the X and Y orthotropy directions were observed: 47.3%, 60.8%, and 54.9% of the strength in the Y direction, and in tropical conditions by 52.7%, 44.4%, and 58.8%, respectively. The relations are very similar also for non-impregnated beams. When using P30, H25, and H20 facing, in dry conditions D, the bending strength of the beams in the direction of X orthotropy is greater by 49.1%, 61.1%, and 52.3%, respectively, concerning the strength in the Y direction, and in tropical conditions W, by 48.5%, 47.8%, and 49.1%, respectively. Therefore, it can be concluded that also in this case, the effect of impregnation on the strength of beams with F-cells is visible. Moreover, the test results illustrate the effect of large and slightly changing orthotropy of the tested beams. Hence, it should be concluded that the effect of impregnation on the change of orthotropic properties of the plate with F-cells is significant.

**Figure 9:**
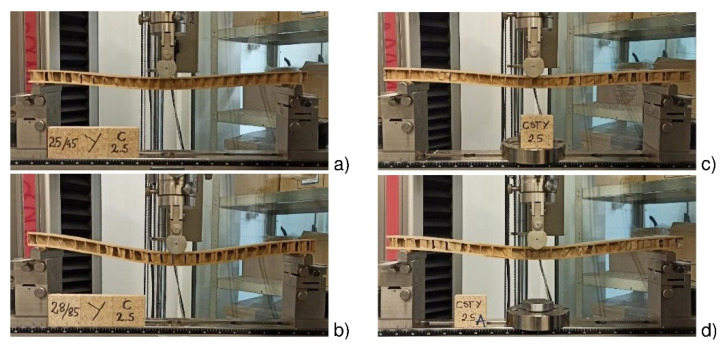
Illustration of the destruction of C-core cell plates: (**a**) CNH25YD beam (**b**) CNH25YW beam (**c**) CSH25YD beam (**d**) CSH25YW beam.

Figure 9 shows deflections of a beam with C-type cells, H25 facings, before and after impregnation (N, S), examined in the direction of the *Y*-axis in the conditions of dry D and tropical W climate. Under tropical conditions (T = 28 °C/H = 85%, Figure 9b), the beam deflection is much higher compared to beams loaded under dry climate conditions (T = 25 °C/H = 45%, Figure 9a). On the other hand, the beams reduce deflections in dry and tropical conditions after impregnating starch, respectively (Figure 9c,d).

### 3.2. Effect of Impregnation on the Panel’s Stiffness

**Figure 10:**
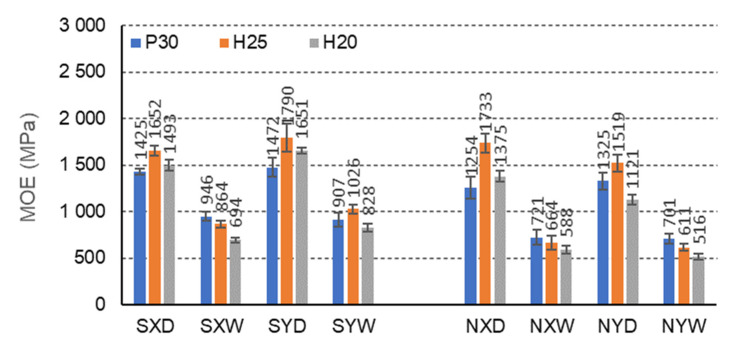
Illustration of the MOE relationship of honeycomb panels with a core of C-cells. Whiskers represent standard deviations.

**Figure 11:**
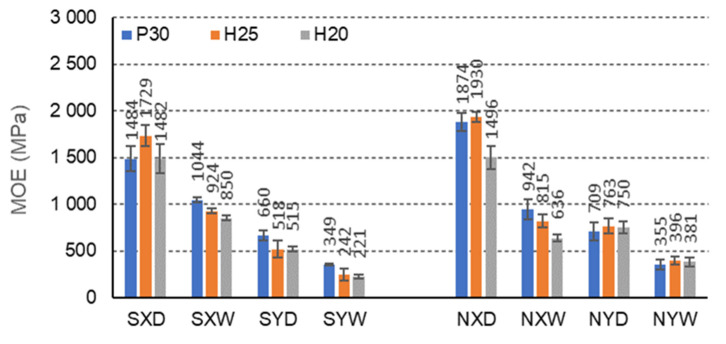
Illustration of the MOE relationship of honeycomb panels with an E-cell core. Whiskers represent standard deviations.

**Figure 12:**
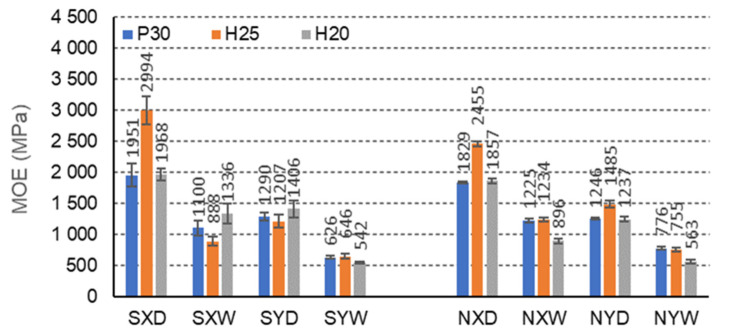
Illustration of the MOE relationship of honeycomb panels with F-type core. Whiskers represent standard deviations.

As shown above, impregnating the core paper with a 10% aqueous acetylated starch water repellant improves the strength of the three-layer furniture panel and its stiffness. This is illustrated in the figures below (Figure 10, Figure 11 and Figure 12). The average increase in the modulus of linear elasticity MOE for all tested beam combinations is approximately 7%.

For C-cell beams, under all climatic conditions, starch impregnation improves the MOE for each case of a facing used and the direction of orthotropy (Figure 10). Only CNH25XD beams with H25 facing in dry conditions and for the X orthotropy direction show a greater modulus of elasticity (1733 MPa) compared to the corresponding beams (CSH25XD) impregnated with starch (1649 MPa). It should be noted that for beams with P30, H25, and H20 facings, in dry conditions D, and for the orthotropy direction X, the MOE increases by 12.0%, −5.1%, and 14.6%, respectively, and in tropical conditions W, respectively by 23.9%, 23.2%, and 15.2%. For beams with the same facings P30, H25 and H20, in dry conditions D, and for the direction of orthotropy Y, the MOE increases by 9.9%, 15.1%, and 32.1%, respectively, and in tropical conditions W by 22.6%, 40.5%, and 37.7%. It is also characteristic that, as in the case of strength changes, no significant difference in linear elasticity modules was observed in the X and Y orthotropy directions for C-type cells. In the orthotropy direction, X is 3.2%, 8.6%, and 10.6% lower concerning the MOE in the Y direction, and in tropical conditions, W is lower by −4.2%, 18.8%, and 19.3%, respectively. Note that a negative value indicates the opposite tendency to decrease, i.e., increase. For non-impregnated beams, these relationships are significantly different. For beams with P30, H25, and H20 facing, in dry conditions D, the MOE in the X orthotropy direction is respectively greater by −5.7%, 12.3%, and 12.1% concerning the MOE in the Y direction, and in tropical conditions W, by 2.6%, 8.0%, and 12.4%, respectively. Thus, the influence of impregnation on the modulus of elasticity of beams with C-cells is visible, but also on the change of this property depending on the direction of orthotropy. After impregnation, the influence of the orthotropy direction on the MOE of the beams changed and diversified. The impregnation of the paper resulted in a weakening of the modulus of linear elasticity of the beams in the direction of the *X*-axis in favor of increasing the MOE in the direction of the *Y*-axis. Although the differences are insignificant, they persuade the orthotropic properties of the honeycomb panels.

In the case of beams with E-cells, the effect of impregnation on the modulus of elasticity is also pronounced (Figure 11). The highest MOE is shown for beams with H25 facing, not impregnated, in dry conditions D, and orthotropy direction X (1930 MPa). In dry conditions D, impregnation with starch significantly reduces the MOE of beams with P30, H25, H20 facings for the X orthotropy direction, compared to analogous non-impregnated beams, by 26.2%, 11.7%, 0.9%, respectively. In tropical conditions, W increases this property by 9.7%, 11.7%, and 25.3%, respectively. For beams with the same facings P30, H25 and H20, in dry conditions D, and for the Y orthotropy direction, MOE also decreases by 7.4%, 47.6%, and 45.4%, respectively, and in tropical conditions W, by 2.0%, 63.6%, and 72.7%. It is also noticeable that clear differences in the modulus of elasticity of the beams in the X and Y orthotropy directions were observed for E-type cells, 55.5%, 70.1%, and 65.2% concerning the MOE in the Y direction, and in tropical conditions by 66.6%, 78.3%, and 74.1%, respectively. For non-impregnated beams, these relationships are very similar. For beams with P30, H25, and H20 facings, in dry conditions D, the modulus of elasticity in the X orthotropy direction is respectively higher by 62.1%, 60.5%, and 49.9% concerning the MOE in the Y direction, and in tropical conditions W, by respectively, 62.3% 51.4%, and 40.2%. In this case, a clear influence of impregnation on the modulus of elasticity of beams with E-cells is visible. The presented results also illustrate the effect of large and changing orthotropy of the tested beams. The changes result both from the use of starch as an impregnating agent and the slender shape of the cells. It is clear that in the case of an elongated E-cell with long free walls (l = 13 mm), impregnation weakens the elastic properties of the core in the direction of the X and Y axes, but at the same time reduces the difference between the modulus of elasticity in these directions.

The F-cell beams also show significant strength differences due to the impregnation of the paper (Figure 12). FSH25XD beams show the highest modulus of elasticity with H25 facing, impregnated, in dry conditions D, and for the orthotropy direction X (2994 MPa). FNH25XD beams achieve much lower MOE with H25 facing, not impregnated, in dry conditions D, and for the same direction of orthotropy (2455 MPa). In dry conditions D, impregnation with starch improves the MOE of beams with P30, H25, H20 facings for the X orthotropy direction, compared to non-impregnated beams by 6.2%, 18.0%, 5.4%, respectively, and in tropical conditions W, by respectively −11.3% 12.5%, and 32.9%. Beams with the same facings P30, H25, and H20, in dry conditions D, and for the direction of orthotropy Y also show a higher MOE by 12.0%, −23.0%, and 12.0%, respectively, and in tropical conditions W, lower MOE by respectively, 24.0%, 17.1%, and 3.9%. There were also visible differences in the modulus of elasticity of the beams in the X and Y orthotropy directions to MOE in the Y direction, and tropical conditions W, by 43.1%, 54.3%, and 59.5%, respectively. The relationships are also similar for non-impregnated beams. When using P30, H25, and H20 facing, in dry D conditions, the MOE of the beams in the X direction is higher by 31.9%, 39.5%, and 33.4%, respectively, concerning the MOE in the Y direction, and in tropical conditions W, by 36.6%, respectively, 38.8% and 37.3%. Therefore, it can be concluded that in this case, the effect of the impregnation on the strength of the beams with F-cells is visible. The changes result both from the use of starch as an impregnating agent and the slender shape of the cells. There is a regularity that in the case of an elongated F cell with short free walls (l = 6.3 mm), the impregnation strengthens the elastic properties of the core in the direction of the *X*-axis. On the other hand, in the direction of the *Y*-axis, the paper’s impregnation contributed to the reduction of the linear elasticity modulus.

### 3.3. Effect of Impregnation on the Energy Absorption

**Figure 13:**
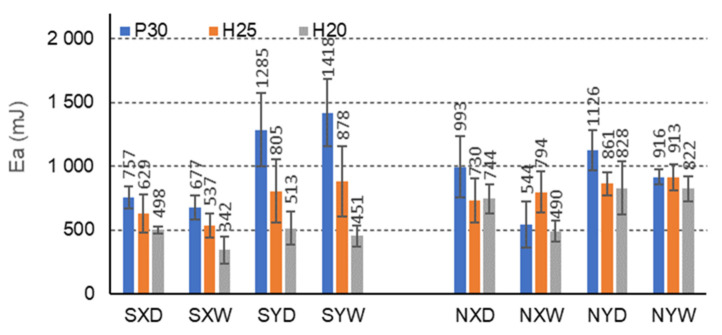
Illustration of the $E_{a}$ relationship of honeycomb panels with a core of C-cells. Whiskers represent standard deviations.

**Figure 14:**
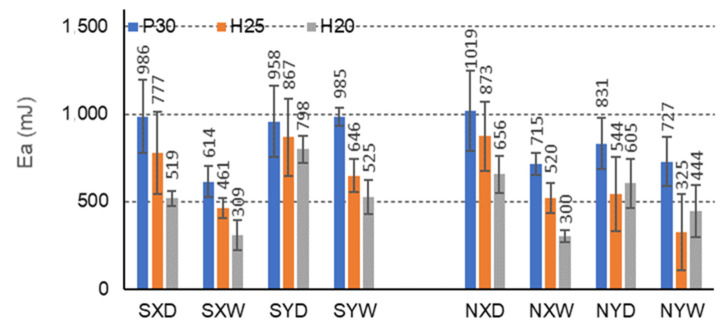
Illustration of the $E_{a}$ relationship of honeycomb panels with an E-type core. Whiskers represent standard deviations.

**Figure 15:**
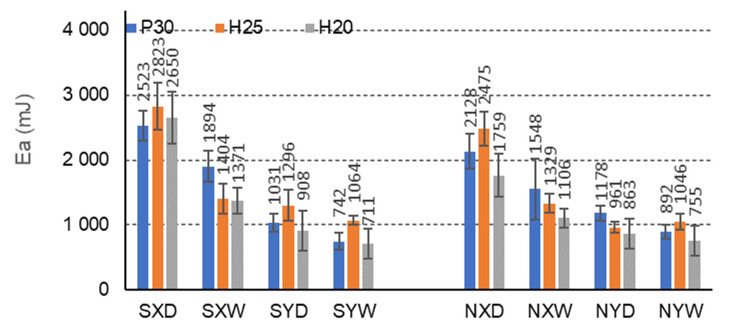
Illustration of the $E_{a}$ relationship of honeycomb panels with F-type core. Whiskers represent standard deviations.

The more energy the composite can absorb, the more effective it is to protect the protected charge. The research (Figure 13, Figure 14 and Figure 15) shows that the FNH25XD and FSH25XD beams have the highest energy absorption capacity before and after impregnation $E_{a}$ = 2474 mJ i $E_{a}$ = 2823 mJ, respectively.

For beams with C-cells, under all climatic conditions, impregnation with starch significantly reduces the amount of energy absorbed for the case of a facing used and the direction of orthotropy (Figure 13). It should be noted that for beams with P30, H25, and H20 facings, in dry conditions D, and for the orthotropy direction X, the amount of absorbed energy decreases by 31.2%, 16.1%, and 49.4%, respectively, and in tropical conditions W, by −19.5%, 47.7%, and 43.3%. For beams with the same facings P30, H25, and H20, in dry conditions D, and for the direction of orthotropy Y, the amount of energy absorbed also decreases by −12.3%, 6.8%, and 61.4%, respectively, and in tropical conditions W, by −35.5, 3.9% and 82.3%, respectively. However, clear differences in the amount of absorbed energy were observed dependent on the X and Y orthotropy directions, 3.0% concerning the amount of energy absorbed in the Y direction, and in tropical conditions W, respectively 109.8%, 63.5%, and 31.9% lower. For non-impregnated beams, these relationships are significantly more favorable. For beams with P30, H25, and H20 facings, in dry conditions D, the amount of absorbed energy in the X direction is respectively lower by 13.4%, 17.8%, and 11.3% about the amount of energy absorbed in the Y direction, and in tropical conditions W, by 68.2%, 15.0%, and 67.8%, respectively. Thus, the effect of impregnation on the reduction in the amount of absorbed energy of beams with C-type cells is visible, but also on the change of this property depending on the direction of orthotropy. The impregnation of the paper caused a reduction in the ability to absorb energy in the X and Y directions. The differences illustrated are significant and convincing to the honeycomb panels’ orthotropic properties in terms of energy absorption.

In the case of beams with E-cells, the effect of impregnation on the amount of energy absorbed is presented in Figure 14. The greatest amount of absorbed energy is shown by beams with P30 facing, not impregnated, in dry conditions D, and for the orthotropy direction X (1019 mJ). In dry conditions D, impregnation with starch significantly reduces the amount of absorbed energy for beams with P30, H25, H20 facings, for the X orthotropy direction, compared to analogous non-impregnated beams by 3.3%, 12.2%, 26.4%, respectively. This property also reduces by 16.5%, 12.8%, and 3.2% in tropical conditions, respectively. For beams with the same facings P30, H25 and H20, in dry conditions D, and for the direction of orthotropy Y, the amount of energy absorbed increases by 13.3%, 37.3%, and 24.2%, respectively, and in tropical conditions W, by 26.1%, 49.8%, and 15.6%. It is also noticeable that for E-type cells, clear differences were observed in the amount of energy absorbed for the beams in the X and Y orthotropy directions; it is, respectively, 2.8%, 11.6%, and 54.1% lower concerning the amount of energy absorbed in the Y direction, and tropical conditions by 60.5%, 40.4%, and 69.9%, respectively. These relationships are different for non-impregnated beams. For beams with P30, H25, and H20 facings, in dry conditions D, the amount of absorbed energy in the X direction is 18.4%, 37.6%, and 7.6% greater, respectively, concerning the amount of energy absorbed in the Y direction, and in tropical conditions W, by −1.8%, 37.6% and −48.2%, respectively. In this case, a variable influence of impregnation on the amount of absorbed energy is visible for beams with E-cells. The changes result both from the use of starch as an impregnating agent and the slender shape of the cells. The regularity is drawn that in the case of an elongated E cell with long free walls (l = 13 mm), the impregnation weakens the core’s ability to absorb energy in the *X*-axis direction and increases this ability in the Y direction.

F-cell beams also show marked differences in the amount of energy absorbed due to the impregnation of the paper (Figure 15). The highest amount of absorbed energy is shown by FSH25XD beams with H25 facing, impregnated, in dry conditions D, and for the orthotropy direction X (2822 mJ). A much smaller amount of absorbed energy is achieved by FNH25XD beams with H25 facing, not impregnated, in dry conditions D, and for the same direction of orthotropy (2475 mJ). In dry conditions D, impregnation with starch improves the amount of absorbed energy for beams with P30, H25, H20 facings, for the X orthotropy direction, about non-impregnated beams by 15.7%, 12.3%, 33.7%, respectively, and in tropical conditions by W, respectively, by 18.3%, 5.4%, and 19.3%. Beams with the same facing P30, H25, and H20, in dry conditions D, and for the orthotropy Y direction, show a reduction in the amount of absorbed energy by 14.3%, –26.0%, and −4.9%, respectively, and in tropical conditions W, a lower amount of absorbed energy by 20.2%, −1.7% and 6.2%, respectively. There were also clear differences in the energy absorbed for the beams in the X and Y orthotropy directions: 65.8% concerning the amount of energy absorbed in the Y direction, and tropical conditions by 60.9%, 24.2%, and 48.2%, respectively. The relationships are also similar for non-impregnated beams. When using P30, H25, and H20 facing, in dry conditions D, the amount of absorbed energy for the beams in the X orthotropy direction is, respectively, 44.7%, 61.2%, and 50.9% greater than the amount of energy absorbed in the Y direction, and in tropical conditions W, by 42.4%, 21.2% and 31.8%, respectively. Therefore, it can be concluded that in this case, impregnation’s effect on the amount of absorbed energy is visible for beams with F-cells. The changes result both from the use of starch as an impregnating agent and also from the slender shape of the cells with short free walls (l = 6.3 mm). The impregnation increases the amount of absorbed energy, especially in the direction of the *X*-axis. On the other hand, in the direction of the *Y*-axis, the impregnation of the paper tended to reduce the amount of absorbed energy.

The observations listed above are generally consistent with the current state of knowledge. For example, Pohl [11] showed in his work that an adequately selected impregnating agent increases the strength (MOR) and stiffness (MOE) of light boards with a paper core. On the other hand, the climatic conditions with high air humidity have been repeatedly quoted in the literature [53,54,58] as having a destructive effect on the mechanical properties of furniture boards. Furthermore, the influence of the geometry, wall thickness, and the orientation of the core cells on the stiffness and strength of lightweight three-layer panels is also known as meaningful [42,68,78].

### 3.4. Statistical Analysis

In order to determine the influence of impregnation and other variable factors on the mechanical properties of the modeled furniture panels, a statistical analysis was performed using the Statistica 13.3 program (StatSoft Polska Sp. z oo, Kraków, Poland).

**Figure 16:**
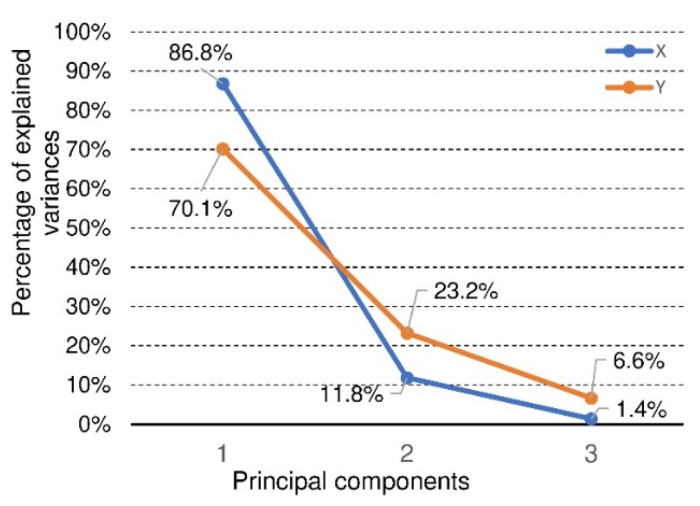
Principal components of feature connected with beams orthotropy in X and Y direction.

The statistical model included five factors (starch impregnation, cell type (C, E, F), facing type (H20, H25, P30), climate condition (D, W), orthotropy direction (X, Y)) and three features (MOR, MOE and $E_{a}$). Because panels with a paper honeycomb core are characterized by strong orthotropy manifested by significant differences in MOR, MOE and $E_{a}$ values for the X and Y directions, the data for statistical analysis was divided into two groups. The first group consisted of factors (starch impregnation, cell type, facing type, and climate condition) and three features (MOR, MOE and $E_{a}$) for the direction of X orthotropy as well as appropriate factors and features for the direction of Y orthotropy. On this basis, an analysis of the correlation of the analyzed features in individual experiments was prepared. The correlation coefficients presented in Table 4 show that the features (MOR, MOE and $E_{a}$) for the X and Y orthotropy directions, respectively, are strongly correlated with each other. Because all the determined correlation coefficients (r) are statistically significant (*p* < 0.05), it was concluded that the further application of the multiple features model to assess the significance of the influence of individual factors on these features could be burdened with some “redundancy”. In this situation, it was justified to use a transformation that would allow us to analyze the influence of all factors differentiating the experiment’s results on all features simultaneously but avoiding their mutual strong linear correlation. For this purpose, principal components analysis was used [79,80], in which three features of MOR, MOE and $E_{a}$ were transformed into three principal components (1), (2), (3), which are their linear combinations (Figure 16).

Figure 16 shows that for further analysis of the significance of the influence of selected factors on three features (MOR, MOE and $E_{a}$) for the direction of X orthotropy, only the first principal component (1) should be left because it explains about 87% of the entire variability in the model. Moreover, in the first component (1), the individual features (MOR, MOE and $E_{a}$) are proportionally represented. In the case of the features from the experiment for the direction of the Y orthotropy, the first two principal components (1), (2) should be taken into account because together they explain more than 93% of the entire variability of the model. The contribution of individual features to the main components is presented in Table 5. It is worth noting that in the experiment for the direction of the Y orthotropy in the second principal component (2), the feature $E_{a}\;$ plays a fundamental role.

Taking into account the above considerations, the tests of the hypotheses about the significance of differences in mean values for the first principal component (1) in the experiment for the direction of orthotropy X (one-way and multivariate ANOVA) and the significance of differences in mean values for the first and second principal components (1), (2) were started, in the experiment for the direction of orthotropy Y (univariate MANOVA). Table 6 shows that the influence of individual factors on the selected main components (1) and (1), (2) for the directions of orthotropy X and Y, respectively, is statistically significant (*p* < 0.05). The HSD Tukey test was performed on this basis, which indicated statistically significant differences in the results for selected variable factors (Table 7 and Table 8).

Table 7 shows that similar mean values of the principal component (1) are obtained for the X orthotropy direction. Therefore, MOR is obtained for E and C cells, and statistically different for an F cell. These differences are clearly visible when comparing Figure 6, Figure 7, Figure 8, Figure 9, Figure 10, Figure 11, Figure 12, Figure 13, Figure 14 and Figure 15. For example, the average MOR value for CSP30XD, ESP30XD, FSP30XD is 4.3 MPa, 5.1 MPa and 11.2 MPa, respectively. Thus, it can be seen that the mean MOR values for boards with E and C cells are similar, and the differences are statistically insignificant. On the other hand, for the F-type cells, the obtained values are statistically different, which also positively and statistically significantly increases both MOR and MOE. At the same time, it can be seen that each type of cell significantly affects the amount of energy $E_{a}$ absorbed by the panels.

Further analysis of Table 7 leads to the following observations. Similar mean values of the principal component (1) were obtained for facing type H25 and P30, and statistically different for facing type H20. As an example, we can give the average MOR values for CSP30XW, CSH25XW, CSH20XW, which are equal to 2.7 MPa, 2.9 MPa and 2.4 MPa, respectively, and for ESP30XW, ESH25XW, ESH20XW or FSP30XW, FSH25XW, FSH20XW boards, respectively 2.9 MPa, 2.6 MPa, and 2.6 MPa, 7.4 MPa, 7.2 MPa and 6.8 MPa. Thus, it can be seen that the average MOR values for panels with H25 and P30 facings are similar, and the differences are statistically insignificant. Moreover, these facings have a positive effect on increasing both MOR and MOE compared to the H20 type facings because in this case, the average values of the principal component (1) are statistically different and lower. The comparison of Figure 13, Figure 14 and Figure 15 also shows that $E_{a}\;$ for panels with H25 facing has higher values than panels with P30 and H20 facing.

Table 7 also shows a statistically significant difference in the mean values of the principal component (1) obtained for tropical climatic conditions (W). A similar regularity applies to the lack of impregnation of the core cells with starch (Y). Because, in both cases, the differences are clearly visible in Figure 6, Figure 7, Figure 8, Figure 9, Figure 10, Figure 11, Figure 12, Figure 13, Figure 14 and Figure 15, it can only be concluded that under dry conditions (D), the MOR values for the cellular plates reach the highest values, similar to the impregnation of paper with starch (S). The beneficial effect of impregnation (S) on the MOR of the tested boards is visible based on the results of tests in dry (D) and tropical (W) conditions. For example, the average MOR value for CSP30XD, ESP30XD, FSP30XD boards is 4.3 MPa, 5.1 MPa, and 11.2 MPa, respectively, and for the boards without impregnation (N), CNP30XD, ENP30XD, FNP30XD, respectively, are 4.2 MPa, 6.3 MPa, and 11.0 MPa. On the other hand, the average MOR value for CSP30XW, ESP30XW, FSP30XW boards is equal to 2.7 MPa, 2.9 MPa, 7.4 MPa, respectively, and for CNP30XW, ENP30XW, FNP30XW boards, 1.8 MPa, 2.5 MPa, and 6.8 MPa, respectively. It can also be seen that the impregnation (Y) of the core cells causes variations in the amount of energy absorbed. Comparison of Figure 13, Figure 14 and Figure 15 shows that $E_{a}\;$ for plates with non-impregnated cells has higher values compared to boards with impregnated cells.

Table 8 shows the statistical significance for the model in a multidirectional classification, therefore taking into account many factors and their interactions. Of course, the values of the first principal component (1) were tested here. It is clear from this table that all factors and their interactions are statistically significant (*p* < 0.05).

Table 9 shows that for the direction of the Y orthotropy, different mean values of the principal components (1), (2) were obtained, thus MOR and the linearly correlated MOE for the selected cell types. For example, the average MOR value for CSP30YD, ESP30YD, FSP30YD is 4.6 MPa, 2.1 MPa, and 5.9 MPa, respectively. The MOE value for the same panels is 1472 MPa, 660 MPa, and 1290 MPa, respectively, and the $E_{a}\;$ value is 1285 mJ, 1959 mJ, and 1031 mJ, respectively. Thus, it can be seen that the average values of MOR, MOE, $E_{a}\;$ for the plates are different and statistically significant.

Moreover, the analysis of Table 9 leads to further observations that similar mean values of component (1) were obtained for facing type H25 and P30 and statistically different for facing type H20. As an example, we can give the average MOR values for CSP30YW, CSH25YW, CSH20YW which are equal to 3.0 MPa, 2.9 MPa, and 2.5 MPa, respectively, and for ESP30YW, ESH25YW, ESH20YW or FSP30YW, FSH25YW, FSH20YW boards, respectively, 1.5 MPa, 1.1 MPa, 1.0 MPa, 3.5 MPa, 4.0 MPa and 2.8 MPa. Different mean values of the component (2) were obtained for the same facings. For example, for the CSP30YW, CSH25YW, CSH20YW, the MOE is 907 MPa, 1026 MPa, and 828 MPa, respectively, and for the ESP30YW, ESH25YW, ESH20YW or FSP30YW, FSH25YW, FSH20YW plates, it is 349 MPa, 242 MPa, 221 MPa, 626 MPa, 646 MPa, and 542 MPa, respectively. Thus, it can be seen that the average MOR values for panels with H25 and P30 facings are similar, and the differences are statistically insignificant. In addition, these facings have a positive effect on increasing both MOR and MOE compared to H20 facings. The comparison of Figure 13 and Figure 14 also shows that $E_{a}\;$ for panels with P30 facing has higher values than panels with H25 and H20 facings.

Table 9 also shows a statistically significant difference in the mean values of the main component (1) obtained for dry climatic conditions (D). A similar pattern applies to the impregnation of core cells with starch (Y). Because, in both cases, the differences are clearly visible in Figure 6, Figure 7, Figure 8, Figure 9, Figure 10, Figure 11, Figure 12, Figure 13, Figure 14 and Figure 15, it can only be concluded that under dry conditions (D), the MOR and MOE values for the honeycomb boards reach the highest values, similar to the impregnation of paper with starch (S). The beneficial effect of impregnation (S) on the MOR and MOE of the tested boards is visible on the basis of the results of tests in dry (D) and tropical (W) conditions. For example, the average MOR value for the CSP30YD, ESP30YD, FSP30YD boards is 4.6 MPa, 2.1 MPa and 5.9 MPa, respectively, and for the boards without impregnation (N), CNP30YD, ENP30YD, FNP30YD, respectively, are 3.2 MPa, 2.0 MPa, and 5.5 MPa. On the other hand, the average MOR value for the CSP30YW, ESP30YW, FSPand FSP30YW boards equals 3.0 MPa, 1.5 MPa, and 3.5 MPa, respectively, and for CNP30YW, ENP30YW, FNP30YW boards, respectively, 1.6 MPa, 1.2 MPa and 3.5 MPa. The MOE value for the same CSP30YW, ESP30YW, FSP30YW and CNP30YW, ENP30YW, FNP30YW panels is equal to 907 MPa, 349 MPa, 626 MPa, 701 MPa, 355 MPa and 776 MPa, respectively. The comparison of Figure 13 and Figure 14 also shows that $E_{a}\;$ for panels with cells impregnated with P30 facings has higher values compared to panels with cells not impregnated with starch.

In order to be able to assess the statistical significance of the factor interactions, only the value of the main component was taken into account in the multidirectional analysis (1). Table 10 shows the statistical significance for the model in the multidirectional classification for the value of only the component (1). It was decided so because, for component (1), we still maintain as much as 70% of the model’s variability (Figure 16). This table shows that only the interactions of Conditions*Starch, Core*Facing*Conditions, Core*Conditions*Starch, Core*Facing*Conditions*Starch are not statistically significant.

## 4. Conclusions

The conducted experiments and the analysis of the results made it possible to determine how the variable above-mentioned factors affect the strength of the three-layer honeycomb panels (MOR), the linear elasticity modulus (MOE), and the ability to absorb energy ($E_{a}$). The most important conclusions and observations are listed below:

- There is a statistically proven significant relationship between the impregnation of paper with modified starch and the mechanical properties of the produced honeycomb panels with variable cell geometry and various types of facing, under varying temperature and relative air humidity conditions.
- In dry conditions (T = 25 °C/H = 45%), the impregnation increases the flexural strength (MOR) of the honeycomb panels by an average of 18% and the linear elasticity modulus (MOE) by 7%. The average ability to absorb energy after starch impregnation increased by 6%.
- In tropical conditions (T = 28 °C/H = 85%), the impregnation increases the flexural strength of the honeycomb panels by an average of 22% and the modulus of linear elasticity by 14%. The average ability to absorb energy after starch impregnation increased by 6%.
- FSH25YD and FSP30YW lightweight panels show the highest flexural strength in dry and tropical conditions.
- FSH25YD and FSH25YW lightweight panels are the stiffest in dry and tropical conditions.
- The most energy in dry and tropical conditions is absorbed by FSH25YD and FSP30YW lightweight panels, respectively.
- F-shaped cells and H25 facings have the best influence on the mechanical properties of the honeycomb panels.
- F-shaped cells and P30 facings most favorably affect the energy absorption capacity of the honeycomb panels.

## Figures and Tables

**Table 1:** Characteristics of cells used for tests, where: ρ —relative cell density, $S_{y}$ —cell width, $L_{x}$ —cell length, l—length of the free cell wall, h—length of the double cell wall, t—thickness of the cell wall (paper), φ –cell wall inclination angle (Figure 1).

Cell Type	ρ	$S_{y}$	$L_{x}$	I	h	t	φ
	(−)	(mm)					(°)
C	0.0249	23.28	20.15	12.2	6.0	0.15	20
E	0.0249	13.33	46.48	13.0	12.0	0.15	60
F	0.0585	9.47	46.84	6.3	19.1	0.25	45

**Table 2:** Physical and mechanical properties of the materials used (MOE—modulus of linear elasticity, MOR—modulus of rupture, ϑ –Poisson’s ratio, $F_{\max}$ —maximal destructive force, X, Y—orthotropy directions, MC—moisture content, SD—standard deviation).

Code	Statistics	Thickness	MC	Density	MOE		MOR		ϑ		$F_{max}$	
		[mm]	[%]	[kg/m^3^]	X	Y	X	Y	XY	YX	X	Y
					[MPa]				-		[N]	
15N	Mine	0.15	5.72	686	5707	2188	46	16	0.411	0.147	105	36
	SD	0.01	-	-	672	113	1.8	0.30	0.043	0.023	4	0.7
15S	Mine	0.16	7.05	730	5190	2642	49	20	0.308	0.109	110	45
	SD	0.02	-	-	374	102	3.1	0.34	0.033	0.010	7	0.8
25N	Mine	0.25	6.11	745	5372	2153	46	17	0.398	0.160	175	67
	SD	0.02	-	-	200	37	1.8	0.50	0.024	0.017	7	2.0
25S	Mine	0.26	6.67	825	4454	2153	45	17	0.348	0.160	170	67
	SD	0.04	-	-	99	37	1.1	0.50	0.021	0.017	4	2.0
P30	Mine	2.77	6.76	942	4116	3445	14	10	0.161	0.129	1539	1085
	SD	0.02	-	18	276	210	2.3	1.50	0.027	0.026	269	171
H25	Mine	2.41	5.28	965	5496	5183	32	31	0.265	0.257	3080	3030
	SD	0.02	-	19	253	164	2.4	2.00	0.024	0.020	228	187
H20	Mine	1.97	5.55	912	4756	4293	22	23	0.243	0.218	1730	1822
	SD	0.02	-	12	324	295	3.5	1.30	0.030	0.031	279	97

**Table 3:** Method of marking samples prepared for testing (The symbol * indicates the selected cell type, impregnation, facing type, orthotropy direction, climate condition).

	Cell Type			Impregnation		Facing Type			Orthotropy Direction		Climate Condition	
Code	*C*	*E*	*F*	*S*	*N*	P30	H25	H20	*X*	*Y*	*D*	*W*
$CSP_{30}XD$	*			*		*			*		*	
$CSP_{30}XW$	*			*		*			*			*
$CSP_{30}YD$	*			*		*				*	*	
$CSP_{30}YW$	*			*		*				*		*
$CNP_{30}XD$	*				*	*			*		*	
$CNP_{30}XW$	*				*	*			*			*
$CNP_{30}YD$	*				*	*				*	*	
$CNP_{30}YW$	*				*	*				*		*
…												
$ESH_{25}YW$		*		*			*			*		*

**Table 4:** The matrix of correlation of features in individual experiments.

Features	MOR		MOE		$E_{a}$	
	r		r		r	
	X	Y	X	Y	X	Y
MOR			0.83	0.77	0.92	0.51
MOE	0.83	0.77			0.65	0.34
$E_{a}$	0.92	0.51	0.65	0.34		

**Table 5:** Contribution of individual features to the principal components.

Features	X	Y	
	1	1	2
MOR	0.37	0.41	0.03
MOE	0.30	0.36	0.24
$E_{a}$	0.33	0.23	0.73

**Table 6:** The significance of the influence of individual factors on the principal components for the directions of X and Y orthotropy.

Factors	p	
	X	Y
Cel type	0.000000	0.000000
Facing type	0.016232	0.000000
Climate condition	0.000000	0.000000
Starch impregnation	0.035712	0.000970

**Table 7:** Summary of statistically homogeneous groups determined on the basis of the HSD Tukey test for selected variable factors in the research for the direction of orthotropy X. (The symbol **** meaning that results are not statistically different).

Variable Factor	Homogeneous Group	
Cel type	a	b
F		****
E	****	
C	****	
Facing type	a	b
H25	****	
P30	****	****
H20		****
Climate condition	a	b
D	****	
W		****
Starch impregnation	a	b
Y	****	
N		****

**Table 8:** Statistical significance for the model in the multidirectional classification takes into account the X orthotropy direction.

Factors and Their Interactions	*p*
Core	0.000000
Facing	0.000000
Conditions	0.000000
Starch	0.000000
Core*Facing	0.000000
Core*Conditions	0.000000
Facing*Conditions	0.000000
Core*Starch	0.000000
Facing*Starch	0.002824
Conditions*Starch	0.009293
Core*Facing*Conditions	0.000000
Core*Facing*Starch	0.000067
Core*Conditions*Starch	0.000000
Facing*Conditions*Starch	0.001572
Core*Facing*Conditions*Starch	0.007842

**Table 9:** Summary of statistically homogeneous groups determined on the basis of the HSD Tukey test for selected variable factors in the research for the direction of orthotropy Y. (The symbol **** meaning that results are not statistically different).

Variable Factor	Homogeneous Group		
Cel type	a	b	c
E	****		
C		****	
F			****
Facing type	a	b	
H20		****	
H25	****		
P30	****		
Climate condition	a	b	
W	****		
D		****	
Starch impregnation	a	b	
N	****		
Y		****	

**Table 10:** Statistical significance for the model in the multidirectional classification taking into account the direction of the Y orthotropy.

Factors and Their Interactions	*p*
Core	0.000000
Facing	0.000000
Conditions	0.000000
Starch	0.000000
Core*Facing	0.000000
Core*Conditions	0.000000
Facing*Conditions	0.019208
Core*Starch	0.000000
Facing*Starch	0.014356
Conditions*Starch	0.661232
Core*Facing*Conditions	0.153976
Core*Facing*Starch	0.000074
Core*Conditions*Starch	0.057297
Facing*Conditions*Starch	0.016343
Core*Facing*Conditions*Starch	0.133444

## Data Availability

The data supporting reported results by the authors can be sent by e-mail.
